# Long-Term In Situ Conservation Drove Microevolution of Solina d’Abruzzo Wheat on Adaptive, Agronomic and Qualitative Traits

**DOI:** 10.3390/plants12061306

**Published:** 2023-03-14

**Authors:** Caterina Morcia, Riccardo De Flaviis, Valeria Terzi, Maria Eugenia Gasparelli, Roberta Ghizzoni, Franz-W. Badeck, Fulvia Rizza, Veronica Santarelli, Giorgio Tumino, Giampiero Sacchetti

**Affiliations:** 1 Consiglio per la Ricerca in Agricoltura e l’Analisi dell’Economia Agraria—Centro di Ricerca Genomica e Bioinformatica (CREA-GB), Via San Protaso 302, 29017 Fiorenzuola d’Arda, Italy; 2 Department of Bioscience and Technology for Food, Agriculture and Environment, University of Teramo, Via R. Balzarini 1, 64100 Teramo, Italy; 3 Plant Breeding, Wageningen Plant Research, Wageningen University & Research, Droevendaalsesteeg 1, 6708 PB Wageningen, The Netherlands

**Keywords:** *Triticum aestivum*, growth habit, in situ conservation, vernalization, allelic variation

## Abstract

Solina is an example of a bread wheat landrace that has been conserved in situ for centuries in Central Italy. A core collection of Solina lines sampled in areas at different altitudes and climatic conditions was obtained and genotyped. A clustering analysis based on a wide SNP dataset generated from DArTseq analysis outlined the existence of two main groups, which, after F_st_ analysis, showed polymorphism in genes associated with vernalization and photoperiod response. Starting from the hypothesis that the different pedoclimatic environments in which Solina lines were conserved may have shaped the population, some phenotypic characteristics were studied in the Solina core collection. Growth habit, low-temperature resistance, allelic variations at major loci involved in vernalization response, and sensitivity to photoperiod were evaluated, together with seed morphologies, grain colour, and hardness. The two Solina groups showed different responses to low temperatures and to photoperiod-specific allelic variations as well as the different morphology and technological characteristics of the grain. In conclusion, the long-term in situ conservation of Solina in environments sited at different altitudes has had an impact on the evolution of this landrace which, despite its high genetic diversity, remains clearly identifiable and distinct so as to be included in conservation varieties.

## 1. Introduction

Solina bread wheat is an ancient landrace which, until recently, was cultivated extensively in Abruzzo, a Central Italian region mainly mountainous (65%) and hilly (34%), with a plain (1%) consisting only of a narrow coastal strip. Starting from the mid-twentieth century, with the spread of highly productive modern varieties, the Solina cultivation area shrank, remaining mainly in marginal areas of high hills and mountains and in areas characterized by low fertility soils. Although its cultivation has been drastically reduced, Solina wheat has continued to be present in the Abruzzo region, ensuring its “in situ” conservation. Such conservation is of particular value for crop genetic resources as the accessions are maintained in a state of continuous interaction with the environment. In “ex-situ” conservation, ensured by germplasm banks, the accessions, preserved as a cyclically renewed seed, are fixed and crystallized with respect to the time of their collection, while in the case of “in situ” conservation, the accessions are in constant evolution.

Solina d’Abruzzo is an exemplary case of “in situ” conservation, thanks to the local farmers who have continued its cultivation over time and thanks to the local demand for traditional products, which specifically require the technological characteristics of Solina flour [1]. Moreover, Solina was extensively used as a parent in Italian breeding programs of the early decades of the twentieth century [2].

The peculiarities of the history and of the agronomic and qualitative characteristics of this wheat have been valorised by the Abruzzo region, which has supported Solina’s registration in the Italian National Register of Conservation Varieties and has promoted various dissemination and research actions.

Landraces are classically made up of a set of lines, with a highly variable level of genetic diversity, depending on the population considered. In this regard, Solina wheat, which was included in a European study aimed at the conservation, use, and study of landraces significant for European cereal farming [3], was found to be characterized by high levels of genetic diversity and by the presence of different within population haplotypes [4]. Such a high genetic diversity is difficult to explain in an autogamous crop; however, the authors speculate that it can be traced back to the past “on-farm” management mode. Significant quantities of Solina were, in fact, cultivated by many farmers, exchanging seeds and thus favouring recombination. In spite of the high level of within-population genetic variability, Solina wheat is a clearly identifiable population, defined as “stable and distinct”, with relatively homogeneous and well-defined phenotypic characteristics.

**Figure 1:**
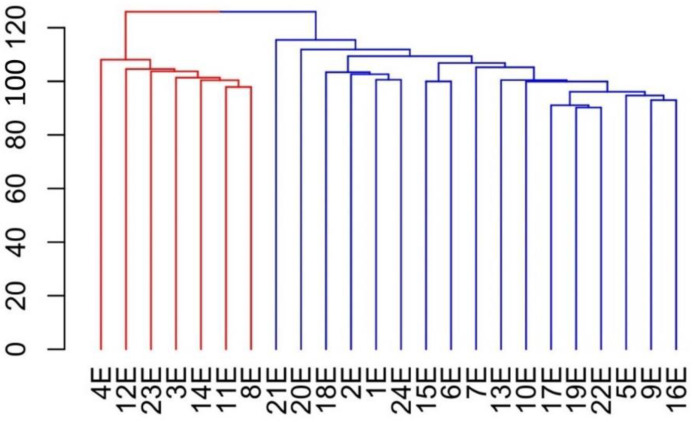
Hierarchically cluster analysis (HCA) based on Euclidean distances and average-linkage agglomeration method computed using a dataset of 15,959 SNP markers, adapted with permission from De Flaviis et al. [5]. Two Solina clusters are highlighted by red and blue colours.

In the work of Khan et al., the characterization of genetic diversity within Solina was accomplished using a small number of single nucleotide polymorphism (SNP) markers and a small number of samples [4]. In a deeper study by De Flaviis et al., a high number of SNP markers were used to evaluate genetic diversity in a panel of Solina samples collected in different Abruzzo farms and located in different areas [5]. The clustering analysis based on these datasets has clearly outlined the existence of two major clusters (Figure 1).

The two genetic groups brought together lines sampled in areas with different climatic conditions, including, for instance, a significant difference in average altitudes. Starting from the hypothesis that the in-situ conservation of Solina in different pedoclimatic environments may have shaped its adaptation, in the present work, Solina lines were characterized for two key adaptive traits, such as growth habitus and resistance to low temperatures and grain quality traits. Allelic variation in the main genes controlling adaptation-related traits has been investigated. In addition, an evaluation of genetic differentiation (F_st_) between the two clusters was carried out to identify highly differentiated genomic regions and possibly link them to known adaptation-related loci.

## 2. Results

### 2.1. Growth Habitus

**Figure 2:**
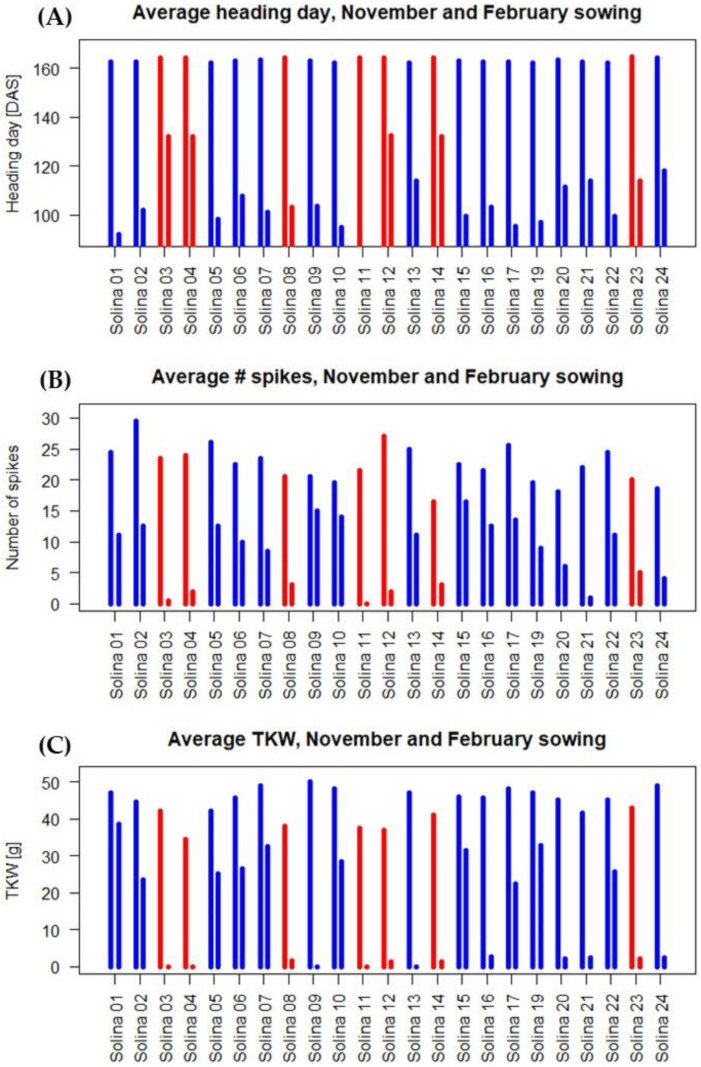
(**A**) Heading date (days after sowing) for November (left bars) and February (right bars) sowing, (**B**) Number of spikes, and (**C**) thousand kernel weight (g) of Solina lines are sown in November (left bars) and February (right bars). Mean values are reported. The colours of the bars indicate membership to the red and blue clusters according to De Flaviis et al. [5].

Solina wheat is traditionally cultivated in autumn sowing. The 23 sub-populations that were sampled in the different geographic areas were sown in the field in autumn 2020 and 2021 and had regularly come to head. In order to verify the actual winter habitus of this wheat, greenhouse trials were carried out at CREA-GB in 2021/2022. All the accessions were sown on three different dates (November, February, and June). The plants were then grown in greenhouses in accordance with the protocols routinely applied in terms of irrigation and fertilization. The heading dates were determined, and, at maturity some agronomic parameters were measured (Figure 2).

In autumn sowing, all accessions reached the heading stage after 162–164 days, whereas no accession flowered when sown in late spring sowing (4 June), as shown in Figure 2A, in which the Solina accessions belonging to the two major clusters distinguished by De Flaviis et al. [5] are indicated with red and blue bars. According to the statistical analysis reported in Table 1, there were no significant differences in the heading date between accessions for November sowing, when the accessions of the red cluster headed slightly later (1.4 days) than the blue cluster. In early spring sowing, a variable number of days was required by the different accessions to flowers (Figure 2A). The range of days required after the 18 February sowing was between a minimum of 93 and a maximum of 132 days. The accessions included in the red cluster required a mean number of 124 days to flower, whereas the accessions of the blue cluster headed significantly earlier (Table 1), after 103 days from sowing. Moreover, Solina 11, included in the red cluster, never flowered after early spring sowing. The joint analyses of relative ranks for the sowing dates with heading plants resulted in a significant population x sowing date interaction and a significant (*p* < 0.001) between-cluster difference.

**Figure 3:**
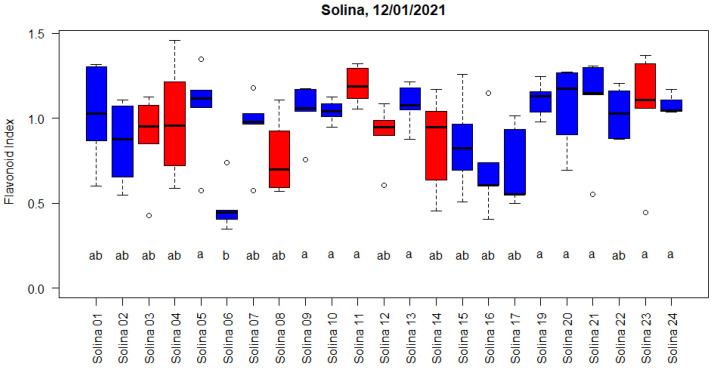
Dualex flavonoid index measured on 12 January 2022, i.e., at DAI 36. The colours of the boxes indicate membership to the red and blue clusters according to De Flaviis et al. [5]. Flavonoid index did not differ significantly for accessions with the same letter according to Kruskal-Wallis test and multiple comparison with Bonferroni correction.

At the end of the life cycle, some agronomic characteristics were assessed, i.e., plant height, above-ground biomass, the number of spikes, and thousand kernel weight (TKW). The results are reported in Figure 3 and Figure S1, from which it can be observed that the different sowing dates shaped the four traits measured.

The mean plant heights were 163 ± 7.8 cm in autumn (10 November) sown plants, 108 ± 21.2 cm in early spring (18 February) sown plants, and 59 ± 8.8 cm in late spring (4 June) sown plants (Figure S1). The standard deviation was higher in the early spring sown class. In this class, Solina accessions of the blue cluster were significantly taller (99.3 cm mean plant height) than the “red” ones (87.7 cm mean plant height) (Table 1). Between accession, differences were significant for all sowing dates, and between cluster differences for November and June sowing dates (Table 1). The joint analyses of relative ranks for all three sowing dates resulted in a significant population × sowing date interaction and a significant (*p* < 0.001) between cluster difference.

The mean biomass production differed significantly between populations for all sowing dates (Table 1) and was 12% higher in the blue cluster than in the red cluster for sowing in November, while for the spring sowing dates, no significant cluster differences were found.

The mean number of spikes was 22 ± 3.1 in November sown plants and 7.9 ± 5.2 in February sown plants, as shown in Figure 2B. No plants sown in June flowered. During February sowing, the accessions belonging to the blue and red clusters differed significantly for this trait, with a mean number of 10.5 spikes for the blue accessions and 2.2 for the red ones, while in November, sowing populations of the blue cluster had only slightly more spikes (+4% not significant) than the populations of the red cluster (Table 1).

The mean TKW of 44 ± 4.2 g was found in autumn sown plants, and 13 ± 14.3 g was found for the February sown ones (Figure 2C). High variability was present for this trait among early spring sown plants (Table 1): the blue Solina accessions had 18.5 g TKW, whereas the red ones had 0.91 g TKW. It is noteworthy that Solina 21, considered an outlier between the red and blue clusters according to De Flaviis et al. [5], in the February sowing resulted phenotypically closer to red accessions than to blue ones for the characters measured (Figure 2C). The joint analyses of relative ranks for the sowing dates with heading plants resulted in a significant population x sowing date interaction and a significant (*p* < 0.001) between cluster difference.

### 2.2. Phenotyping for Frost Tolerance and Other Leaf Traits

Out of 15 imbibed seeds, ten or more germinated until the days after imbibition (DAI) 11 for all populations. The average Dualex chlorophyll (Chl; 24.3 and 26.7) and flavonoid index values (Flav; 0.52 and 1.10), as well as the nitrogen balance index (NBI; 53.2 and 28.9), were measured at DAI 21 and DAI 48, respectively, and did not significantly differ between accessions. Epidermal flavonoid content, indicated by the flavonoid index, increased between the first measurement at DAI 21, before cold acclimation, and DAI 48, seventeen days after the change to acclimation temperatures of 3/1 °C (Figures S2 and S3). At DAI 36, five days after the change to acclimation temperatures of 3/1 °C, transient significant differences between the accessions were found (Figure 3). Chl, Flav, and NBI did not differ between genetic clusters except for DAI 48, when the red cluster had, on average, a 13% higher Flav index and 13% lower NBI, while Chl did not differ between the clusters.

The maximal quantum yield of photosystem II (PSII), F_v_/F_m_ measured on dark-adapted leaves at DAI 41, 66, 76, and 91 (i.e., after 10, 35, 45, and 60 days of acclimation at 3/1 °C) did not significantly differ between the populations. Population median values were always above 0.73, indicating healthy, photosynthetically competent leaves. The grand mean varied in the course of time between 0.785, 0.786, 0.765, and 0.777 at DAI 41, 66, 76, and 91, respectively.

**Figure 4:**
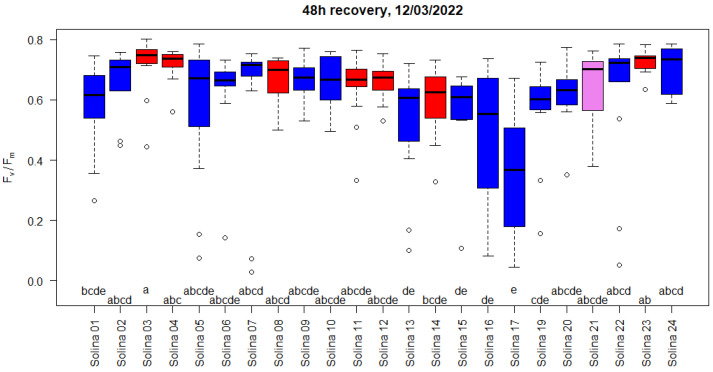
F_v_/F_m_, indicator of freezing damage to PSII, measured after freezing stress at −14 °C and 48h of recovery under acclimation conditions. Experiment on whole plants. The colour of the boxes indicates membership to the red and blue clusters according to De Flaviis et al. [5], with Solina 21 belonging to the blue cluster closest to the red cluster shown in purple. F_v_/F_m_ did not differ significantly for accessions with the same letter according to Kruskal-Wallis test and multiple comparison with Bonferroni correction.

F_v_/F_m_ re-measured after freezing stress and recovery indicated only a generally slight to moderate impairment of PSII ( Figure S4, S5 and Figure 4).

**Figure 5:**
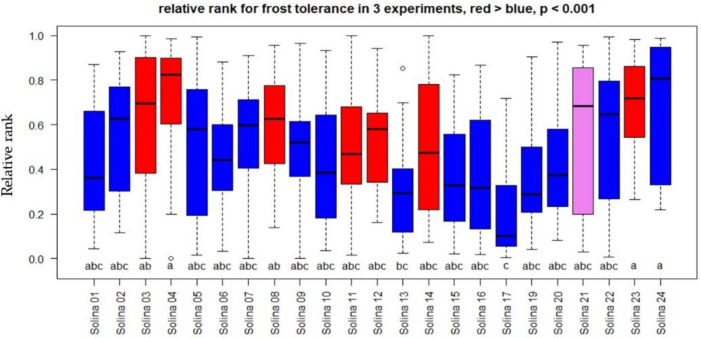
Relative rank of frost tolerance in three freezing stress experiments at −14 °C. The colour of the boxes indicates membership to the red and blue clusters according to De Flaviis et al. [5], with Solina 21 belonging to the blue cluster closest to the red cluster shown in purple. Relative ranks did not differ significantly for accessions with the same letter according to Kruskal-Wallis test and multiple comparison with Bonferroni correction.

The statistical power of the first two experiments (n = 3) was relatively low, resulting in some or no statistically significant differences between accessions except for the first experiment, where accession six was significantly more damaged than accessions 14, 21, and 24. The experiment on whole plants (8 < n < 13) better discriminated between more and less susceptible plants. No plant mortality was observed up to six days after the end of the freezing treatment. The relatively high frost tolerance after freezing stress of −14 °C was in accordance with what could be expected for the species *T. aestivum*. We hypothesize that the lethal temperature (LT_50_) should be close to −15 °C. Relative ranks of F_v_/F_m_ were calculated experiment-wise in order to combine the results of the three experiments. In the two experiments on cut leaves, the population in the red genetic cluster, together with Solina 21, had significantly higher frost tolerance than the accessions in the blue group (*p* < 0.02). Considering that the relative ranks of all three experiments together (Figure 5) resulted in significantly higher frost tolerance of the red group compared to the blue group (*p* < 0.001), Solina 21 was not statistically different from both. Solina accession 17 resulted significantly more susceptible compared to Solina 4, 23, and 24.

At the start of the hardening treatment (DAI 23), the second leaf was still expanding. The leaf length of leaves 1 and 2 measured at DAI 31 did not differ significantly between accessions nor between the red and blue genetic clusters for any of the two leaf ranks. The length of the first leaf was significantly correlated with the length of the second leaf (r = 0.77, *p* < 0.001). Additionally, for the six accessions measured at DAI 52, the width of leaf 2 did not differ significantly between populations, as well as the Haun stage, which was 3.5 on average, i.e., the fourth leaf was expanding. At this time, the accessions demonstrated between 1.4 and 1.8 visible tillers. At DAI 69 and 76, no significant difference in leaf length was found between the genetic clusters. The same held true for the leaf width measured at DAI 76. In conclusion, the accessions and genetic groups exhibited similar phenology and leaf dimensions during the acclimation treatment. Under hardening conditions, the plants still grew slowly.

### 2.3. Allelic Variation at Major Loci Governing Vernalization Response and Photoperiod Sensitivity

Allelic variations in the genes determining vernalization requirement and photoperiod sensitivity have a key role in wheat growth habits and environmental adaptability. The polymerase chain reaction (PCR) based assays reported in the Material and Methods section, have been exploited to explore allelic diversity in Solina accessions (Table 2). Two major polymorphisms were found among Solina accessions. Eight out of twenty-three Solina accessions carried the *vrn-A1b* allele, whereas the other 15 Solinas had *vrn-A1*. A second polymorphism was found in the *VRN-B1* locus: two accessions had the *Vrn-B1a* allele, whereas all the other Solinas had *vrn-B1*.

Chip digital PCR assays were applied to determine the copy number variation affecting the Vernalization-A1 gene, which was found by Diaz et al. to be associated with an altered flowering time in the wheat [6]. The assay developed by Diaz et al. targeting the *Vrn-A1* sequence was coupled with the assay developed by Morcia et al. to target the *Pinb-D1* single-copy gene as an internal positive control [7]. Solina 1, Solina 6, and Solina 7 have two copies of the target gene, whereas all the other Solina have one copy. Within the Solina 21 accession, both seeds with one copy of the target gene, as well as seeds with two copies, were present.

**Figure 6:**
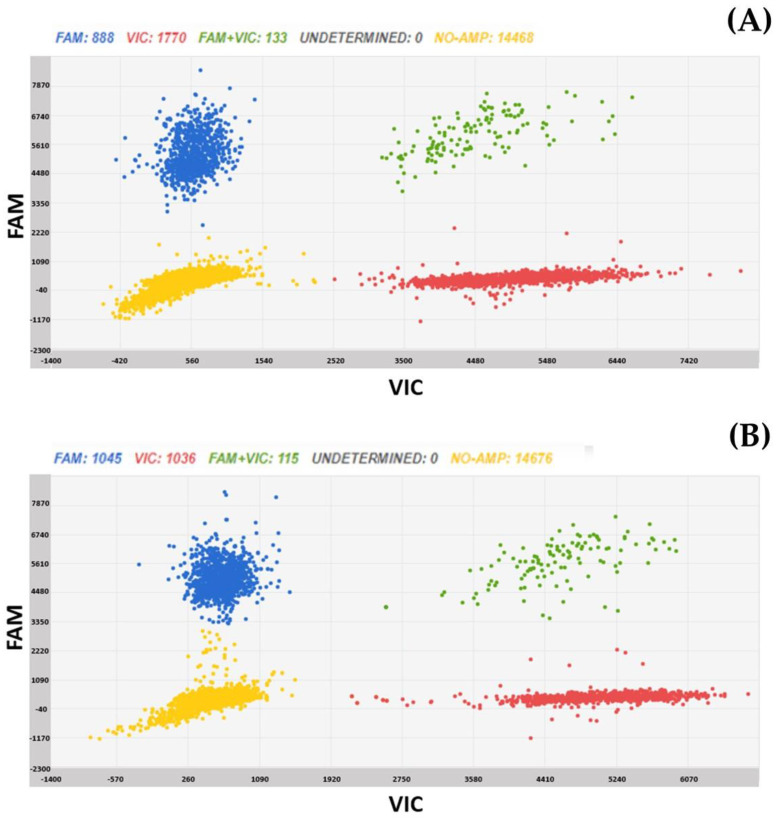
Chip digital PCR scatter plots of Solina 1 (**A**) and of Solina 2 (**B**) samples, carrying, respectively two and one copy of vrn-A1 gene. Blue dots are chip wells in which Pinb-D1 gene (marked with FAM) has been amplified, red dots are wells in which vrn-A1 gene (marked with VIC) has been amplified, green dots are wells in which both genes have been amplified, whereas yellow dots are wells without any amplification. From raw data, the software calculates the number of copies/µL of each gene. The ratio between vrn-A1 copies and Pinb-D1 copies is 1.9 for Solina 1 and 1 for Solina 2.

Two scatter plots of the samples showing a copy number variance (CNV) are shown in Figure 6.

### 2.4. Genetic Differentiation of Clusters by F_st_

**Figure 7:**
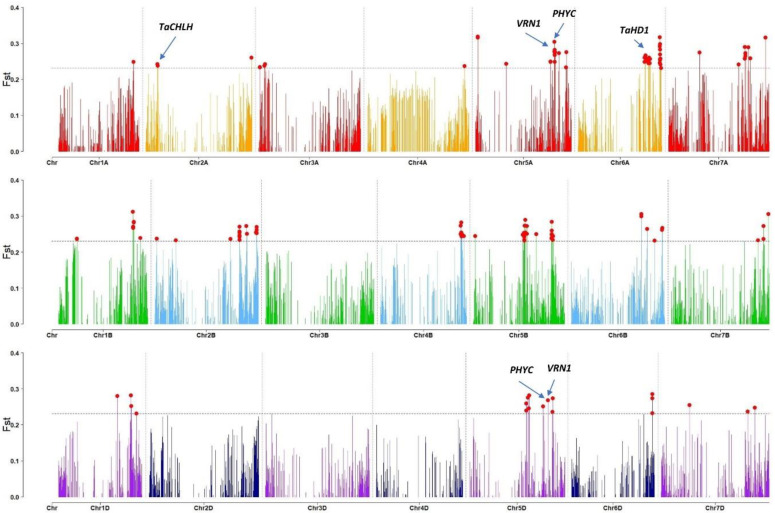
Rectangular Manhattan plot of F_st_ distribution calculated between the red/blue clusters. The dashed horizontal lines indicate the 99 percentiles of F_st_ values. F_st_ values in the highest 1% of the distribution are indicated by red dots and key genes overlapping with extreme F_st_ are labelled.

The genetic differentiation between the “red” and “blue” Solina genetic groups was evaluated by F_st_ using previously developed DArTseq data [5]. F_st_ values were calculated for each marker individually and plotted according to their genomic position (Figure 7). The permutation-based significant threshold was too liberal, resulting in more than 10% of the markers being significantly differentiated. Hence, we opted for an arbitrarily chosen more stringent threshold (the 99th percentile of F_st_ values) to highlight genomic regions with extreme F_st_ values. A total of 147 SNPs were found in the highest 1% of F_st_ values, some of them clustering together in a few genomic regions (Figure 7, in red). The main clusters of markers with extreme F_st_ values were found in Chr1B (6 SNPs at 582.0–588.5 Mb), Chr2A (3 SNPs at 78.0–89.5 Mb), Chr2B (9 SNPs at 665.0–667.0 Mb and 3 SNPs at 799.0–800.5 Mb), Chr4B (9 SNPs at 626.0–636 Mb), Chr5A (9 SNPs at 557.0–591.0 Mb), Chr5B (9 SNPs at 396.5–409.0 Mb and 9 SNPs at 613.0–625.0 Mb), Chr5D (5 SNPs at 340.5–357.0 Mb), Chr6A (5 SNPs at 494.5–501.5 Mb, 9 SNPs at 523.0–534.0 Mb and 11 SNPs at 605.5–616.0 Mb), Chr6D (3 SNPs at 482.5–484.0 Mb), and Chr7A (5 SNPs at 565.0–571.0 Mb).

About 50% of those 147 markers were located in 5A, 5B, 5D, and 6A chromosomes. The co-localization between those regions identified by F_st_ and genes controlling adaptive traits (e.g., *VRN*, *PPD*, *EPS*, *RHT*, and *CBF/DREB* gene families) was investigated. Interestingly, some of the signals in Chr5 co-localized with *VRN-A1* and *VRN-D1*: two major genes controlling vernalization and flowering in wheat (Figure 7). The *PHYTOCHROME C (PHYC)* genes, which regulate genes associated with photoperiod sensitivity and light perception (*PPD1* and *TaHD1*), are very close to the *VRN* genes (Figure 7). The cluster of high F_st_ SNPs on Chr6A (523–534 Mb) co-localized with *TaHD1*: a light-sensitive flowering activator. The clusters in Chr5B (396.5–409.0 Mb) and Chr5D (340.5–357.0 Mb) represent homeologous chromosomic regions, possibly indicating the presence of a common underlying gene under selection. However, we failed to identify in those regions any gene that was related to adaptive traits. Lastly, the gene *TaCHLH* (involved in the light-dependent chlorophyll accumulation) was found in the proximity of the region identified on Chr2A (78.0–89.5 Mb).

### 2.5. Commercial Quality Parameters

Morphological traits of kernels such as the length, width, and thickness (L1, L2, and L3, respectively), dimension ratios (L1/L2 and L1/L3), volume (V), colour, and hardness were analysed on 24 Solina lines (see Table 3 and Table 4 for the ANOVA results).

Within the 24 Solina accessions, the effect of the genetic cluster (individual or interactive) was significant (*p* < 0.05) for all of the analysed parameters except L1/L3 and the L* colour parameter, even though the nested farm effect always resulted in the main effect (*p* < 0.001). The blue cluster was the one with the highest kernel volume (40.7 vs. 36.9 mm^3^), the highest kernel length (7.05 vs. 6.84 mm), width (3.30 vs. 3.13 mm), and thickness (3.34 vs. 3.28 mm), as well as the lowest L1/L2 value (2.14 vs. 2.19), which describes kernel elongation. The blue cluster showed a lower redness (a*) value (8.41 vs. 8.59), which, in turn, was reflected in a higher hue angle (h°) value (69.5 vs. 68.9). The kernels of the blue cluster also showed a higher hardness (55.7 vs. 54.5).

In the sample set obtained with the in-situ experiment (Table 5 and Table 6), the blue cluster, which in a previous study resulted in higher protein content [5], was the one with the highest kernel width (3.22 vs. 3.06 mm) and lowest thickness (3.14 vs. 3.23 mm), as well as the lowest kernel elongation (2.21 vs. 2.32).

**Figure 8:**
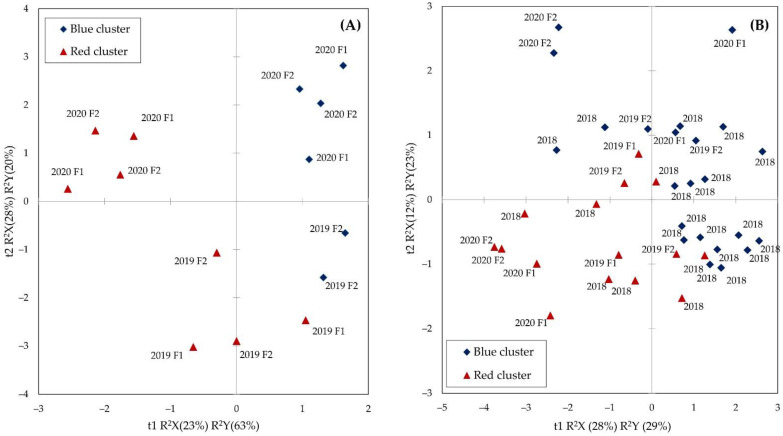
Score plot of the accessions belonging to the two different genetic clusters (red/blue) on the two latent variables (t) calculated by PLS-DA. (**A**) Accessions #2 (blue) and #3 (red) cultivated in the in situ experiments; (**B**) 24 accessions collected in 2018 added with the #2 and #3 accessions cultivated in situ.

By merging the morphological and quality parameter data obtained in the in situ study with those (protein, TKW, and TW) obtained in previous work and by computing a PLS-DA analysis, it was possible to discriminate the two genetic clusters with a 100% correct classification (Figure 8A). The most important variables for classification (VIP > 0.8) were: L1/L3, L3, L1/L2, L2, TW, hardness, TKW, and the hue angle in order of importance.

The samples belonging to the red cluster fell within a wide range along both the t1 and t2 dimensions, whilst the sample of the blue clusters varied mostly along the second dimension (t2), which has a lower R^2^Y value. The second dimension was permitted to differentiate the samples in two years of in situ experiments. The red cluster was more affected by the year of harvesting than the blue one.

By merging the 24 accessions collection and the in situ trial datasets and computing a further PLS-DA, it was possible to discriminate the two genetic clusters with an overall 92% correct classification (Figure 8B), the 100% correct classification of the blue cluster, and 80% classification of the red one. The most important variables for classification (VIP > 0.8) were the morphological traits: L1/L3, L3; L2, TW, TKW, volume, and L1/L2, in order of importance.

## 3. Discussion

Solina d’Abruzzo is a bread wheat landrace that has been in situ conserved for centuries in a relatively restricted region of Central Italy. Starting from the hypothesis that such long-term in situ conservation has the potential to shape the adaptive, agronomic, and quality traits of the landrace, a Solina line collection was created [5]. The samples were collected in farms located in a relatively restricted area, characterized by a strong gradient of altitude and temperature. The climate of Abruzzo is greatly conditioned by the Apennines, which clearly separates the climate of the coastal strip and of the sub-Apennine hills from that of the higher inland mountain strips. The coastal areas have a typical Mediterranean climate with hot, dry summers and mild, rainy winters, thenthe temperatures decrease progressively with altitude. Towards the interior areas, the climate gradually became more continental and eventually a typical mountain climate, especially in the province of L’Aquila. Here, the frosts are frequent and intense, with the presence of snow on the ground and particularly intense cold spells. In this territory, Solina cultivation has been present at least since the sixteenth century [5]. It can be hypothesized that during this long time of “on farm” conservation, recombination events, together with the fixation of neutral and adaptive variants, have shaped Solina accessions, even if its distinctness as a landrace is still undoubtable. Additionally, a selection pressure operated by the farmers on the quality and technological characteristics of the grains can be hypothesized.

The Solina collection has been previously genotyped, and two major genetic clusters were identified [5], as reported in Figure 1. The blue Solina accessions were collected from farmers who cultivated them at the average altitude of 700 m a.s.l., which is common for Solina cultivation, whilst the red accessions were collected from farmers who cultivated them in a confined zone localized in the south-eastern part of the cultivation area whose average altitude was 880 m a.s.l.

Starting from the hypothesis that the different climatic microenvironments, combined with the long period of in situ conservation, could have favoured the emergence of specific allelic variants, the genomic regions with extreme F_st_ values when contrasting between red and blue Solina clusters were studied since they could reflect genomic regions that have undergone selective pressure for phenotypes related to adaptation. We found some of those regions to be co-localizing with major genes affecting flowering in wheat, such as *VRN-1A*, *VRN-1D*, and *TaHD1*. This is in agreement with the allelic variation found in *VRN* genes and with the heading dates observed by sowing Solina lines in February. While these findings represent a good indication that adaptive traits may have been differentially selected between the two Solina clusters, it cannot be excluded that other traits were involved in this process. Yet, for several genomic regions identified by F_st,_ we could not find any co-localizing known gene related to adaptive traits. Additionally, F_st_ is not a formal test of differential selection, and its pattern may be affected by other phenomena, such as, for instance, chromosomic rearrangements.

Wheat has the capacity to grow in very different environments. Several mechanisms contribute to this feature, among which structural and functional variants of gene sequences controlling complex adaptive characters are of great relevance, such as flowering time in response to vernalization, photoperiod, and temperature. The heading date is, in fact, a major determinant of global adaptability in cereals [8,9,10]. The overall flowering pathway is very complex, as reviewed by Kamran et al., with vernalization (*Vrn*) and photoperiod (*Ppd*) as the major genetic systems involved [11]. The wheat growth habit is controlled by genes that can be sensitive or insensitive to vernalization, defined as the “acquisition or acceleration of the ability to flower by a chilling treatment”. Allelic variations in such genes can modulate the vernalization requirement, resulting in spring, facultative, or winter habits. During the photoperiod impact on the transition from the vegetative to reproductive phase, spikelet initiation, and stem elongation processes compete for assimilate supply. Ppd alleles are critical for the duration of this transition, with an impact on fertility and yield [11].

Based on the results obtained, it can be concluded that all the Solina accessions are winter types: their transition from a vegetative to reproductive phenological phase is completed after autumn sowing, and all the plants reach full maturity. On the contrary, late spring sowing inhibits this transition, and all plants remain in the vegetative stage. An intermediate situation has been observed after early spring sowing: some of the accessions conclude the transition, whereas, in others, the transition is only sketchy. Interestingly, the vernalization request highlighted by February sowing is different between the two clusters (Figure 1), into which Solina accessions can be grouped according to previous genetic analysis [5]. Solina accessions belonging to the red cluster require a higher level of vernalization to leave the vegetative phase in comparison to accessions belonging to the blue cluster. Line 21, which is an outlier between the two clusters, is closer to the red accessions for this phenotypic trait.

In the greenhouse environment, the earlier heading of the accessions of the blue cluster was associated with more vigorous growth (higher plant height and biomass), whereas when the vegetative growth phase of the blue cluster accessions was much shorter than that of the red cluster, lower biomass was observed. Eventually, the blue cluster accessions produced more spikes and showed a higher TKW, and thus invested much more in grain production. The accessions of the red cluster had significantly higher frost tolerance in comparison to those of the blue cluster, whereas Solina 21 was intermediate between them.

The markers for *Vrn* and *Ppd* genes confirm the winter growth habit of the Solina landrace. However, some variability has been found in the allelic status of *Vrn* genes among Solina accessions, whereas no variation has been observed for *Ppd* genes. The accessions of the blue cluster carry *vrn-A1*, a wild-type allele encoding for a MADS-box transcription factor expressed in leaves and the shoot apical meristem, whereas the *vrn-A1b* allele is carried by the accessions of the red cluster. Despite the presence of polymorphisms between the two allelic forms, both are involved in conferring winter habitus [12,13]. The *Vrn-1* gene has been evaluated even in terms of copy number variation, using a double digital PCR assay, targeting a reference *Pinb-D1* single-copy gene [7] and *Vrn-A1* gene [6]. The doubling of copies was observed in Solina 1, 6, and 7. This same analysis conducted on Solina 21 single seeds showed the co-presence of seeds with one and two copies.

Finally, all the accessions are monomorphic for *Ppd1* alleles, and all carry the combination *Ppd-A1a.1*, *Ppd-B1b*, and *Ppd-D1b*. The dominant alleles of *Ppd* genes (indicated with the suffix “a”) induce insensitivity to day length and, therefore, accelerate flowering in wheat [14]. In particular, *Ppd-D1a*, the dominant allele of *Ppd-D1,* is a major source of photoperiod insensitivity in wheat cultivars worldwide, whereas its recessive allele, *Ppd-D1b*, inhibits earlier ear emergence and flowering [14,15,16,17]. *Ppd-D1a* confers the earliest flowering time, followed by *Ppd-A1a* and *Ppd-B1a* [18]. Nishida et al. compared the heading time among DH lines differing in the *Ppd*-1 genotype and showed that the effect of *Ppd-A1a* was weaker than that of *Ppd-B1a* or *Ppd-D1a* [19]. The Solina landrace carries two of the *Ppd* genes in the recessive allelic form (i.e., *Ppd-B1b* and *Ppd-D1b*), which are related to sensitivity to day length and the third gene, *Ppd-A1a.1*, in the dominant form, relates to the insensitivity of day length. Seki et al. indicated in *Ppd-A1a* is a useful source for fine-tuning the heading time [20]. However, the interaction of this allele with the other photoperiod-related genes and with vernalization genes is still not clear.

The alleles of *VRN-A1* explained 52% of the deviance in heading dates for sowing in February (GLM). Adding a copy number of *VRN-A1* did only marginally improve the fit, but the model based on the alleles alone was the more parsimonious evaluated with the Akaike Information Criterion. The same was true for height, with an explained deviance of 40.8%, while the number of spikes and TKW were more parsimoniously explained by the alleles and copy number of *VRN-A1,* with an explained deviance of 59.8% and 38.1%, respectively. The contribution of the copy number was always minor.

The increase in epidermal flavonoids during cold acclimation is consistent with the C/N allocation hypothesis that predicts increased allocation to secondary metabolites that do not contain nitrogen, such as in flavonoids when a surplus of carbon is produced relative to its use for the growth of new organs. Under cold acclimation temperatures, growth is reduced more severely than photosynthesis leading to a surplus of carbohydrates. Alternatively, a slowly proceeding exhaustion of nitrogen availability in the limited soil volume of the culture trays could be the cause of the same mechanism. The transient between population differences during the first days of acclimation with Solina 6 significantly lag behind Solina 5, 9, 10, 11, 13, 19, 20, 21, 23, and 24 and indicates a putative difference in the speed of the acclimation response. Additionally, the small reduction in the maximal quantum yield of photosynthesis during the early cold acclimation of population 6 (Fv/Fm = 0.796) lagged behind the average of these populations (Fv/Fm = 0.784).

Genotypic differences among the Solina accessions of the red and blue cluster, which have adapted to different altitudes and hence, climatic conditions, resulted in phenotypic variations that are of interest for their handling, grading, processing, and storage. Phenotypic variation among Solina accessions was already observed by Bonvicini; thus, De Flaviis and coworkers started to study differences in grades, determining factors for the commercial quality of the two groups (protein content, TKW, and TW), but no information was reported on their morphology, colour, and hardness [5,21].

Moisture and colour are grade-determining factors, whilst hardness and morphological characteristics are of interest for handling and processing, including sieving and milling, in particular. The Solina accessions of the red cluster, which have adapted to higher altitudes, are morphologically different from those of the blue cluster, and they generally show lower L1, L1/L2 values, higher a*, and lower hue angle values, which denote a redder hue. The kernel shape and the size difference are visible to the unaided eye, and the colour difference between the two groups is above the just notable difference (JND) value (2.3) and could be easily perceived by the sight.

By merging morphological, colour, and hardness data with those of protein content, TW, and TKW, it was possible to clearly discriminate between Solina accessions belonging to the blue and red clusters. The accessions of the red cluster, which were adapted to high altitudes, showed higher annual variability in morphometric and quality traits than those of the blue cluster independent of the experimental fields.

The Solina accessions of the red cluster showed a higher elongation of the kernels, lower kernel dimensions, lower hardness, lower protein content, and TKW (data previously reported) than those of the blue cluster; these characteristics are less recommendable for grading and processing purposes, even though they did not show a significant difference in agronomic yield [5]. This latter trait, in particular, was a key factor for the agriculture practiced in marginal areas located at high altitudes, and favoured the use of the Solina landrace over centuries, as well as its in situ conservation.

The results obtained within this study can lead to some considerations. The first is that the genetic diversity enclosed in a landrace is the engine for the evolution of variants with greater resilience and environmental adaptability. Bocci et al. suggested that an “evolutionary population grown continuously in different locations evolved into locally adapted populations with significant differences in important quantitative traits” [22]. Even landraces, whose definition changed over time, are evolutionary populations in a constant state of change driven by natural and artificial selection, as pointed out by Casanas et al. [23].

Solina variability can be considered the result of a long-term, unwitting experiment in which environmental and human factors impact its microevolution, with the development of variants for both adaptive and qualitative characteristics. This population is, therefore, an interesting genetic material that can deepen our knowledge of the evolution and conservation of genetic resources.

The second consideration is that Solina has an important cultural value for farmers and local communities because of its qualitative peculiarities and traditional uses. The value of landraces for in situ conservation and valorisation have recently been discussed by Raggi et al. [24], with the aim of creating an inventory for the entirety of Europe. The idea to protect a landrace and foster its use has been pioneered by Solina d’Abruzzo.

## 4. Materials and Methods

### 4.1. Plant Materials

A collection of 24 accessions of Solina wheat was sampled by the University of Teramo in different locations representative of the area where Solina was grown, as previously reported by De Flaviis et al. [5]. The collection sites were all in Abruzzo, mainly distributed in the province of L’Aquila, except for two genotypes that were collected in the Pescara province (Table 7).

The average altitude at which the harvest took place was 755 ± 263 above sea level, with different pedoclimatic conditions typical of the Abruzzo region. The 24 accessions were sampled at local farms where Solina wheat was grown for at least ten years under organic conditions. Accession 18 was not included in the greenhouse trial, and the subsequent frost tolerance test and DNA extraction were due to germination issues. Even in a repeated germination trial after 12 days of stratification at 3/1 °C, Solina 18 did not germinate.

### 4.2. Greenhouse Trial

Seeds of all the Solina accessions were sown in 20 cm diameter pots in a greenhouse without any light and temperature adjustments with respect to the external environment on subsequent dates (10 November 2021; 18 February 2022; 4 June 2022). Five seeds/pots were sown for each accession in duplicate. The pots were regularly watered and fertilized, and the heading dates were registered. At full maturity, the following parameters were determined: plant heights (cm), above-ground plant biomass (g), number of spikes, and Thousand Kernel Weight (g). As conditions for the application of ANOVA were not met for most of the traits, a non-parametric Kruskal–Wallis test was applied to test the Null hypothesis of equal trait values between accessions or HCA clusters with subsequent multiple comparisons for applying the Bonferroni correction.

### 4.3. Frost Tolerance Test

Seeds of 23 Solina landraces were used for phenotyping. Fifteen seeds per landrace were imbibed on 7 December 2021 and kept in a growth cabinet at 20/15 °C day/night.

At 11 days after imbibition, the plantlets were transplanted to culture trays. On 30 December (DAI 23) and 7 January (DAI 31), the temperature in the growth cabinet was lowered to 12/7 °C and 3/1 °C day/night, respectively, in order to acclimate the plants to low temperatures.

Measurements of leaf chlorophyll (Chl), epidermal flavonoids (Flav), and a nitrogen balance index (NBI) were taken before the start of hardening (DAI 21) and during hardening (DAI 36 and DAI 48), i.e., after 5 and 17 days of hardening at 3/1 °C.

Three third leaves per landrace were harvested for every two frost tolerance tests at 66 and 76 DAI, respectively, and were kept in falcons (see [25] for validation of the method). A third test for frost tolerance was performed on all plants within the cultivation trays at DAI 91. A maximum photosystem II quantum yield, F_v_/F_m,_ was measured after dark adaptation on the fourth leaf. Subsequently, the leaves or plants were exposed to a frost stress treatment with temperatures gradually decreasing down to −14 °C, which was kept for several hours and then gradually increased again to 1 °C. F_v_/F_m_ was again measured directly after the end of stress and after 24 h or 48 h of recovery within the growth cabinet at 3/1 °C in the experiments on cut leaves and plants, respectively.

The leaf lengths of the first and second leaves were measured at 31 DAI. Leaf width and phenology were assessed on 28 January 2022 for six populations (Solina 19 through 24). The leaf length of the third leaf was measured at DAI 69 and DAI 76 on the leaves used for the frost tolerance tests of cut leaves. At DAI 76, the leaf width of the third leaf was measured.

### 4.4. DNA Extraction and PCR Based Assays

Young leaf tissues were sampled, and their genomic DNA was extracted using a DNeasy Plant Mini Kit (Qiagen, Milan, Italy). Both bulked (derived from 5 plantlets) and single-seed derived plantlets were sampled. The evaluation of the quality and quantity of the extracted DNA was performed using a Qubit™ fluorometer in combination with the Qubit™ dsDNA BR Assay kit (Invitrogen by Thermo Fisher Scientific, Monza, Italy).

End-point PCR reactions were performed with the primers reported in Table 8.

The following PCR protocol was used: 30 ng genomic DNA in 20 µL reactions comprising 1× PCR buffer and 0.2 µL Taq polymerase (GoTaq Flexi, Promega Corp., Milano, Italy) with 3 mM MgCl_2_, 250 µmol of each primer and 0.5 mM dNTPs, with 40 cycles of denaturing (95°C for 40 sec), annealing (54–62 °C for 40 s), and polymerization (72 °C for 1 min). The amplification reactions were conducted in an Applied Biosystem 2720 Thermal Cycler (Thermo Fisher Scientific, Monza, Italy) and separated by electrophoresis on 1% agarose gels using a 0.5× TBE (Tris-Borate-EDTA) buffer, visualized under UV light.

A chip digital PCR was performed using a QuantStudioTM 3D Digital PCR System (Applied Biosystems by Life Technologies, Monza. Italy). The primers and probes reported in Table 9 were used. The reaction was conducted in a final volume of 16 µL, which was obtained by mixing 8 µL of QuantStudioTM 3D Digital PCR 2X Master Mix, 0.72 µL of each primer at 20 µM (final concentration 900 nmol), 0.32 µL of FAM, and VIC-MGB probes at 10 µM (final concentration 200 nmol), 2 µL of DNA (20 ng/µL) and nuclease-free water. Nuclease-free water as a template was used in the negative control. The reaction mixture of 15 µL was loaded onto the QuantStudioTM 3D DigitalPCR chips using a QuantStudioTM 3D Digital chip loader, according to the manufacturer’s instructions. Amplifications were performed in ProFlexTM 2Xflat PCR System Thermocycler (Applied Biosystems by Life Technologies, Monza, Italy), and the amplification conditions used are reported in Table 10. End-point fluorescence data were collected in QuantStudioTM 3D Digital PCR Instrument, and files generated were analysed using the cloud-based platform QuantStudioTM 3D AnalysisSuite dPCR software, version 3.1.6. Each sample was analysed in duplicate. The CNV was calculated as the ratio between copies/µL of *Vrn-A1* and copies/µL of *Pinb-D1.*

### 4.5. F_st_ Analysis

The genetic differentiation between populations was analysed using the genotypic dataset developed by De Flaviis et al. [5]. The dataset consisted of 15,959 markers obtained by (DarTseq) genotyping four single seeds per each of the 24 Solina accessions. As a measure of differentiation across the genome, fixation index (F_st_) was estimated, contrasting the variance between the two Solina genetic clusters [30,31]. F_st_ was estimated for each SNP by the function *basic.stats* of the *hierfstat* R package [31,32,33,34]. A significance threshold for F_st_ values was calculated by permuting the assignment of accessions to the two clusters. For each permutation, the maximum F_st_ value was stored to generate a null distribution of values. A total of 100 permutations were performed. In addition, and in accordance with Esvelt Klos et al. [34], a more stringent threshold (the 99th percentile of F_st_ values) was used to identify the most highly differentiated genomic regions only. The *CMplot* R package [32,35] was used to graph the rectangular Manhattan plot.

### 4.6. In Situ Trials

Seeds of accessions #2 and #3 (Table 7) belonging to the blue and red genetic clusters and supplied by Az. Agricola Cipolla, (Castelvecchio Subequo, Italy) and Az. Agricola De Santis (Introdacqua, Italy), respectively, were used for field trials conducted in randomized plots in the Abruzzo region (Italy).

The experimental fields were located in two different farms coded as F1 and F2 and sited in Corropoli (42°49′ N, 13°51′ E, 70 m a.s.l.) and Castelvecchio Subequo (42°07′ N, 13°44′ E, 500 m a.s.l.), respectively; they presented different soil and climatic characteristics. The two accessions were cultivated in the 2018/2019 and 2019/2020 seasons. The sowing was performed between the end of October and the beginning of December 2018 and 2019 in a 500 m length plot area. All accessions were collected between the beginning and middle of July 2019 and 2020 depending on altitude. During this period, the phytosanitary quality of the crops was periodically checked.

### 4.7. Commercial Quality Parameters

The phenotyping of several commercial quality parameters was carried out. The moisture content was also analysed (AACC 44–15.02). The total nitrogen content (NC) was determined by the Kjeldahl method (AACC 46–12.01), and protein content was calculated using a conversion factor of 5.7. Test weight (TW) and thousand-kernel weight (TKW) were analysed after removing all impurities. All analyses were carried out in duplicate.

Size and shape were evaluated on 30 kernels using a Vernier caliper (to 0.01 mm). The principal dimensions of the major (L1), intermediate (L2), and minor (L3) diameters, volume, and internal ratios (L1/L2 and L2/L3) were measured and calculated. The volume of the seed was then calculated using Miller’s (1987) equation: V (cm^3^) = π (L1*L2*L3)/6.

The colour of kernels was measured in bulk by a Minolta (Osaka, Japan) colorimeter model CR5 equipped with the D65 illuminant and using the 10° standard observer. L*, a*, b*, C*, h°, and ΔE were measured. Twenty repetitions of colour analysis were carried out. Grain hardness was measured in bulk by NIR analysis (AACC 39–70.02), and two repetitions were carried out.

### 4.8. Statistical Analyses

Between population differences in frost, tolerance were assessed with a Kruskal–Wallis test with Bonferroni correction for multiple comparisons (R 2019, version 3.5.3) [32]. This non-parametric test was applied for the analysis of F_v_/F_m_ and relative ranks as they were confined within the [0, 1] interval and were generally non-normally distributed [36]. For other traits, the Kruskal–Wallis test was applied if conditions for ANOVA were not met.

The statistical analyses of quality parameters were performed using XLSTAT 2021 software (Addinsoft, Paris, France). Mixed nested ANOVA was carried out on data from the 24 accessions of Table 7 by considering the Clu1/Clu2 as a fixed effect and the farm collection as the random nested effect. Repeated measurement of ANOVA was carried out on data of accessions 2 and 3 (representative of Clu1 and Clu2, respectively) cultivated in farms F1 and F2 for two consecutive years, and the plot and year were used as random effects.

PLS-DA was computed using all the morphological and quality parameters (including those measured by De Flaviis et al. [5]) of the 24 accessions, and the final number of latent variables (LVs) was chosen using the minimum predicted residual error sum of squares (PRESS) approach in cross-validation. A variable importance in projection (VIP) was used as a selection index in order to investigate the most important variables capable of discriminating the two genetic clusters (blue/red). A threshold value of 0.8 was selected to discuss the most important ones.

## 5. Conclusions

Solina d’Abruzzo is a local population of common wheat that has been cultivated for a very long time in an environment that, although restricted, has peculiar environmental and climatic variations. It is a case of long-term in situ conservation on-farm, able to drive microevolution of the crop under a dynamic management of its diversity [9,37,38]. Both natural and farmers-driven selective forces can, in fact, modulate adaptive, agronomic, and qualitative traits. The case of the Solina landrace is an exemplificative of this microevolutionary phenomenon.

Genome-wide genetic analysis has outlined how the Solina d’Abruzzo landrace can be split into two clusters that correspond to areas at different altitudes and climates. A deeper genetic and phenotypic characterization of the two clusters showed diversity for key adaptive traits that are able to modulate the spreading of a crop [12,13,39,40]. The high-altitude cluster not only has a higher level of vernalization requirement, photoperiod response, and frost resistance but even greater phenotypic variability and greater rusticity and, therefore, an increased ability to respond to environmental stress. As opposed to this, the lower-altitude cluster had higher qualitative and technological properties and traits verisimilarly subjected to positive selection by the farmers for centuries. In conclusion, this study showed how the long-term cultivation of a landrace in a limited area but with varied climatic characteristics could impact the microevolution of the population as a whole with the appearance of lines that have peculiar physiological and genetic variants. Starting from the fact that agrobiodiversity is a pillar of food security, making production systems more resilient [41,42], the genetic diversity harboured by landraces can justify their conservation and cultivation. In particular, Solina d’Abruzzo can be considered the result of a long-term experiment in which biological, edaphic, human, and climatic factors shaped the genetic structure and a number of agronomic and qualitative characteristics that can, therefore, be equated to an evolutionary population. Solina d’Abruzzo has been characterized in the present study for some aspects of its genetic and physiological variability and for a few allelic variants. The results that were obtained demonstrated that this landrace is an interesting genetic resource with open opportunities to deepen our knowledge of the role of further allelic variants resulting from its microevolution.

## Figures and Tables

**Table 1:** Analyses of statistical differences between accessions and genetic clusters. Significance of Kruskal–Wallis tests of null hypothesis of equal trait values between accessions (chi squared accessions) and between genetic clusters (chi squared genetic cluster). The last two columns report the difference and the ratio of the trait values of the blue minus/over the red cluster.

Trait	Sowing Time	Accessions χ^2^ Test (*p* Value)	Genetic Cluster (Blue/Red) χ^2^ Test (*p* Value)	Difference Blue/Red	Ratio Blue/Red
Height	November	<0.027	<0.001		1.05
	February	<0.027	<0.001		1.06
	June	<0.009	0.641		1.00
Biomass	November	<0.004	<0.009		1.12
	February	<0.004	0.189		0.91
	June	<0.008	0.369		0.95
Heading date	November	0.998	<0.032	−1.42	
	February	<0.006	<0.001	−21.06	
spikes ^1^	November	<0.005	0.524		1.13
	February	<0.007	<0.001		4.74
TKW ^2^	November	<0.034	<0.001		1.19
	February	<0.004	<0.001		20.42

^1^ # spikes: number of spikes; ^2^ TKW: thousand kernel weight.

**Table 2:** Allelic variants of vernalization and photoperiod sensitivity genes identified in Solina accessions.

Accession	*VRN-A1*	*VRN-B1*	*VRN-B3*	*VRN-D1*	*PPD-A1*	*PPD-B1*	*PPD-D1*
Solina1	*vrn-A1*	*vrn-B1*	*vrn-B3*	*vrn-D1a*	*Ppd-A1a.1*	*Ppd-B1b*	*Ppd-D1b*
Solina2	*vrn-A1*	*vrn-B1*	*vrn-B3*	*vrn-D1a*	*Ppd-A1a.1*	*Ppd-B1b*	*Ppd-D1b*
Solina3	*vrn-A1b*	*vrn-B1*	*vrn-B3*	*vrn-D1a*	*Ppd-A1a.1*	*Ppd-B1b*	*Ppd-D1b*
Solina4	*vrn-A1b*	*vrn-B1*	*vrn-B3*	*vrn-D1a*	*Ppd-A1a.1*	*Ppd-B1b*	*Ppd-D1b*
Solina5	*vrn-A1*	*vrn-B1*	*vrn-B3*	*vrn-D1a*	*Ppd-A1a.1*	*Ppd-B1b*	*Ppd-D1b*
Solina6	*vrn-A1*	*vrn-B1*	*vrn-B3*	*vrn-D1a*	*Ppd-A1a.1*	*Ppd-B1b*	*Ppd-D1b*
Solina7	*vrn-A1*	*vrn-B1*	*vrn-B3*	*vrn-D1a*	*Ppd-A1a.1*	*Ppd-B1b*	*Ppd-D1b*
Solina8	*vrn-A1b*	*vrn-B1*	*vrn-B3*	*vrn-D1a*	*Ppd-A1a.1*	*Ppd-B1b*	*Ppd-D1b*
Solina9	*vrn-A1*	*Vrn-B1a*	*vrn-B3*	*vrn-D1a*	*Ppd-A1a.1*	*Ppd-B1b*	*Ppd-D1b*
Solina10	*vrn-A1*	*vrn-B1*	*vrn-B3*	*vrn-D1a*	*Ppd-A1a.1*	*Ppd-B1b*	*Ppd-D1b*
Solina11	*vrn-A1b*	*vrn-B1*	*vrn-B3*	*vrn-D1a*	*Ppd-A1a.1*	*Ppd-B1b*	*Ppd-D1b*
Solina12	*vrn-A1b*	*vrn-B1*	*vrn-B3*	*vrn-D1a*	*Ppd-A1a.1*	*Ppd-B1b*	*Ppd-D1b*
Solina13	*vrn-A1*	*vrn-B1*	*vrn-B3*	*vrn-D1a*	*Ppd-A1a.1*	*Ppd-B1b*	*Ppd-D1b*
Solina14	*vrn-A1b*	*vrn-B1*	*vrn-B3*	*vrn-D1a*	*Ppd-A1a.1*	*Ppd-B1b*	*Ppd-D1b*
Solina15	*vrn-A1*	*vrn-B1*	*vrn-B3*	*vrn-D1a*	*Ppd-A1a.1*	*Ppd-B1b*	*Ppd-D1b*
Solina16	*vrn-A1*	*vrn-B1*	*vrn-B3*	*vrn-D1a*	*Ppd-A1a.1*	*Ppd-B1b*	*Ppd-D1b*
Solina17	*vrn-A1*	*vrn-B1*	*vrn-B3*	*vrn-D1a*	*Ppd-A1a.1*	*Ppd-B1b*	*Ppd-D1b*
Solina19	*vrn-A1*	*vrn-B1*	*vrn-B3*	*vrn-D1a*	*Ppd-A1a.1*	*Ppd-B1b*	*Ppd-D1b*
Solina20	*vrn-A1*	*vrn-B1*	*vrn-B3*	*vrn-D1a*	*Ppd-A1a.1*	*Ppd-B1b*	*Ppd-D1b*
Solina21	*vrn-A1b*	*vrn-B1*	*vrn-B3*	*vrn-D1a*	*Ppd-A1a.1*	*Ppd-B1b*	*Ppd-D1b*
Solina22	*vrn-A1*	*Vrn-B1a*	*vrn-B3*	*vrn-D1a*	*Ppd-A1a.1*	*Ppd-B1b*	*Ppd-D1b*
Solina23	*vrn-A1b*	*vrn-B1*	*vrn-B3*	*vrn-D1a*	*Ppd-A1a.1*	*Ppd-B1b*	*Ppd-D1b*
Solina24	*vrn-A1*	*vrn-B1*	*vrn-B3*	*vrn-D1a*	*Ppd-A1a.1*	*Ppd-B1b*	*Ppd-D1b*

**Table 3:** Results of the ANOVA performed on morphometric data in the 24 Solina accessions.

24 Accessions	Type	L1	L2	L3	V	L1/L2	L1/L3
Cluster	Fixed	<0.001	<0.001	<0.001	<0.001	0.006	ns
Farm (Cluster)	Random	<0.001	<0.001	<0.001	<0.001	<0.001	<0.001

ns: not significant.

**Table 4:** Results of the ANOVA performed on commercial quality data in the 24 Solina accessions.

24 Accessions	Type	L*	a*	b*	C*	h°	Hardness	Protein ^1^	TKW ^1^	TW ^1^
Cluster	Fixed	ns	<0.001	<0.001	0.003	<0.001	0.005	ns	0.018	0.017
Farm (Cluster)	Random	<0.001	<0.001	<0.001	<0.001	<0.001	<0.001	<0.001	<0.001	<0.001

^1^ Data from De Flaviis et al. [5]. ns: not significant.

**Table 5:** Results of the ANOVA performed on morphometric data from the in situ experiment.

Effect	Type	L1	L2	L3	V	L1/L2	L1/L3
Plot	Random	ns	ns	ns	ns	ns	ns
Year (Y)	Random	0.003	<0.001	<0.001	<0.001	0.035	0.028
Farm (F)	Fixed	ns	ns	0.016	0.016	ns	ns
Cluster (C)	Fixed	ns	0.004	0.047	ns	0.013	ns
Y × F	Random	ns	ns	0.049	ns	ns	ns
Y × C	Random	ns	ns	ns	ns	ns	0.029
F × C	Fixed	0.006	ns	ns	ns	0.027	0.001

ns: not significant.

**Table 6:** Results of the ANOVA performed on commercial quality data from the in situ experiment.

Effect	Type	L*	a*	b*	C*	h°	Hardness	Protein ^1^	TKW ^1^	TW ^1^
Plot	Random	ns	ns	ns	ns	ns	ns	ns	ns	ns
Year (Y)	Random	ns	<0.001	<0.001	<0.001	ns	0.013	0.008	ns	<0.001
Farm (F)	Fixed	<0.001	ns	ns	ns	0.003	ns	0.004	<0.001	ns
Cluster (C)	Fixed	ns	ns	ns	ns	ns	ns	0.004	0.006	<0.001
Y × F	Random	0.041	ns	0.009	0.013	ns	0.001	<0.001	<0.001	<0.001
Y × C	Random	0.014	ns	ns	ns	0.007	ns	0.011	0.006	ns
F × C	Fixed	ns	ns	ns	ns	ns	ns	ns	ns	<0.001

^1^ Data from De Flaviis et al. [5]. ns: not significant.

**Table 7:** Solina accessions used in the study. Each accession is identified by a number and the sampling location is reported, together with geographical information.

N.ID	Location	Latitude	Longitude	Altitude (m a.s.l.)
1	Castelvecchio Subequo	42.13	13.73	492
2	Castelvecchio Subequo	42.13	13.73	492
3	Introdacqua	42.01	13.90	652
4	Scanno	41.90	13.88	1004
5	Luco dei Marsi	41.96	13.47	664
6	Goriano Sicoli	42.08	13.77	712
7	Tagliacozzo	42.07	13.25	736
8	Rosciolo dei Marsi	42.12	13.34	902
9	Magliano dei Marsi	42.08	13.36	707
10	Magliano dei Marsi	42.08	13.36	707
11	Scurcola Marsicana	42.06	13.34	698
12	Rocca Pia	41.93	13.98	1067
13	Scurcola Marsicana	42.06	13.34	698
14	Pescosansonesco	42.25	13.88	532
15	Farindola	42.44	13.82	514
16	Cagnano Amiterno	42.46	13.23	844
17	Castel Del Monte	42.37	13.73	1354
18	Montereale	42.53	13.24	911
19	Capestrano	42.27	13.77	501
20	Barisciano	42.33	13.59	948
21	Capitignano	42.52	13.30	908
22	Ofena	42.33	13.76	521
23	Rivisondoli	41.87	14.07	1309
24	Elice	42.52	13.97	249

**Table 8:** PCR assays used to genotype Solina accessions for alleles at major vernalization and photoperiod loci.

Primer Name	Primer Sequence [5′–3′]	References
*VRN1AF* *VRN1R*	GAAAGGAAAAATTCTGCTCG TGCACCTTCCC(C/G)CGCCCCAT	[26]
*Intr1/A/F2* *Intr1/A/R3*	AGCCTCCACGGTTTGAAAGTAA AAGTAAGACAACACGAATGTGAGA	[27]
*Intr1/C/F* *Intr1/AB/R*	GCACTCCTAACCCACTAACC TCATCCATCATCAAGGCAAA	[27]
*Intr1/B/F* *Intr1/B/R3*	CAAGTGGAACGGTTAGGACA CTCATGCCAAAAATTGAAGATGA	[27]
*Intr1/B/F* *Intr1/B/R4*	CAAGTGGAACGGTTAGGACA CAAATGAAAAGGAATGAGAGCA	[27]
*VRN4-B-INS-F* *VRN4-B-INS-R*	CATAATGCCAAGCCGGTGAGTAC ATGTCTGCCAATTAGCTAGC	[28]
*VRN4-B-NOINS-F* *VRN4-B-NOINS-R*	ATGCTTTCGCTTGCCATCC CTATCCCTACCGGCCATTAG	[28]
*TaPpd-A1prodelF* *TaPpd-A1prodelR3*	CGTACTCCCTCCGTTTCTTT AATTTACGGGGACCAAATACC	[19]
*TaPpd-A1prodelF* *TaPpd-A1prodelR2*	CGTACTCCCTCCGTTTCTTT GTTGGGGTCGTTTGGTGGTG	[19]
*TaPpd-B1proinF1* *TaPpd-B1proinR1*	CAGCTCCTCCGTTTGCTTCC CAGAGGAGTAGTCCGCGTGT	[19]
*Ppd-D1_F1* *Ppd-D1_R2*	ACGCCTCCCACTACACTG CACTGGTGGTAGCTGAGATT	[29]
*Ppd-D1_F1* *Ppd-D1_R1*	ACGCCTCCCACTACACTG GTTGGTTCAAACAGAGAGC	[29]

**Table 9:** Primers and probes sequences and references are reported. The VRN-A1 assay was used to check *VRN-A1* copy number variation. The TriAPX assay targets a single copy *Triticum aestivum* sequence *Pinb-D1*.

Oligo Name	Sequence (5′ to 3′)	5′ Dye	3′ Quencer	Reference
*TriAPX-prob*	AGCTCTTGCAAGGAT	FAM	MGB	[7]
*TriAPX-For*	AGGAGCGGCCGAAGCT			
*TriAPX-Rev*	TGTGAAACATCGCTCCATCAC			
*VRN-A1*	TGTGTTCGCTTTGGTTGTGCAGGCA	VIC	MGB	[6]
*VRN-A1-For*	GCAGCCCACTTTTGGTCTCTA			
*VRN-A1-Rev*	TCTGCCCTCTCGCCTGTT			

**Table 10:** Amplification conditions used in digital-PCR.

Steps	Temperature	Time	Cycles
Activation	96 °C	10 min	1
Denaturation	98 °C	30 s	47
Annealing/extension	56 °C	2 min	
Final extension	60 °C	2 min	1
Storage	4 °C	∞	1

## Data Availability

The data presented in this study are available on request from the corresponding author.

## Supplementary Materials

The following supporting information can be downloaded at: https://www.mdpi.com/article/10.3390/plants12061306/s1, Figure S1: A, Plant height [cm], B, Biomass [g], bars from left to right correspond to sowing in November, February, and June, the color of the bars indicates membership to the red and blue clusters according to De Flaviis et al. [5]; Figure S2: Dualex flavonoid index measured at 28 December 2021, i.e., at DAI 21; Figure S3: Dualex flavonoid index measured at 24 January 2022, i.e., at DAI 48; Figure S4: F_v_/F_m_, an indicator of freezing damage to PSII, measured after freezing stress at −14 °C and 24 h of recovery under acclimation conditions. The first experiment was on cut leaves. The color of the boxes indicates membership to the red and blue clusters, according to De Flaviis et al. [5], with population 21 belonging to the blue cluster but closest to the red cluster shown in purple; Figure S5: F_v_/F_m_, an indicator of freezing damage to PSII, measured after freezing stress at −14 °C and 24 h of recovery under acclimation conditions. The first experiment was on cut leaves. The color of the boxes indicates membership to the red and blue clusters according to De Flaviis et al. [5], with population 21 belonging to the blue cluster but closest to the red cluster shown in purple.

## References

[1] Porfiri O., Silveri D. (2004). La “Solina” e altre varietà locali di cereali ancora coltivate in Abruzzo: I risultati di una campagna di collezione e caratterizzazione promossa dall’ARSSA. Atti VI Convegno Nazionale Biodiversità.

[2] Piergiovanni A.R. (2013). Evaluation of genetic variation and grain quality of old bread wheat varieties introduced in north-western Italian environments. Genet. Resour. Crop Evol..

[3] Dawson J.C., Serpolay E., Giuliano S., Schermann N., Galic N., Chable V., Goldringer I. (2012). Multi-trait evolution of farmer varieties of bread wheat after cultivation in contrasting organic farming systems in Europe. Genetica.

[4] Khan A.R., Goldringer I., Thomas M. (2020). Management Practices and Breeding History of Varieties Strongly Determine the Fine Genetic Structure of Crop Populations: A Case Study Based on European Wheat Populations. Sustainability.

[5] De Flaviis R., Tumino G., Terzi V., Morcia C., Santarelli V., Sacchetti G., Mastrocola D. (2022). Exploration of the Genetic Diversity of Solina Wheat and Its Implication for Grain Quality. Plants.

[6] Díaz A., Zikhali M., Turner A.S., Isaac P., Laurie D.A. (2012). Copy Number Variation Affecting the *Photoperiod-B1* and *Vernalization-A1* Genes Is Associated with Altered Flowering Time in Wheat (*Triticum aestivum*). PLoS ONE.

[7] Morcia C., Bergami R., Scaramagli S., Ghizzoni R., Carnevali P., Terzi V. (2020). A Chip Digital PCR Assay for Quantification of Common Wheat Contamination in Pasta Production Chain. Foods.

[8] Comadran J., Kilian B., Russell J., Ramsay L., Stein N., Ganal M., Shaw P., Bayer M., Thomas W., Marshall D. (2012). Natural variation in a homolog of *Antirrhinum CENTRORADIALIS* contributed to spring growth habit and environmental adaptation in cultivated barley. Nat. Genet..

[9] Enjalbert J., Dawson J.C., Paillard S., Rhoné B., Rousselle Y., Thomas M., Goldringer I. (2011). Dynamic management of crop diversity: From an experimental approach to on-farm conservation. Comptes Rendus Biol..

[10] Goldringer I., Prouin C., Rousset M., Galic N., Bonnin I. (2006). Rapid Differentiation of Experimental Populations of Wheat for Heading Time in Response to Local Climatic Conditions. Ann. Bot..

[11] Kamran A., Iqbal M., Spaner D. (2014). Flowering time in wheat (*Triticum aestivum* L.): A key factor for global adaptability. Euphytica.

[12] Muterko A., Kalendar R., Salina E. (2016). Allelic variation at the VERNALIZATION-A1, VRN-B1, VRN-B3, and PHOTOPERIOD-A1 genes in cultivars of *Triticum durum* Desf. Planta.

[13] Strejčková B., Milec Z., Holušová K., Cápal P., Vojtková T., Čegan R., Šafář J. (2021). In-depth sequence analysis of bread wheat VRN1 genes. Int. J. Mol. Sci..

[14] Worland A.J., Korzun V., Röder M.S., Ganal M.W., Law C.N. (1998). Genetic analysis of the dwarfing gene Rht8 in wheat. Part II. The distribution and adaptive significance of allelic variants at the Rht8 locus of wheat as revealed by microsatellite screening. Theor. Appl. Genet..

[15] González F.G., Slafer G.A., Miralles D.J. (2005). Pre-anthesis development and number of fertile florets in wheat as affected by photoperiod sensitivity genes *Ppd-D1* and *Ppd-B1*. Euphytica.

[16] Wilhelm E.P., Boulton M.I., Al-Kaff N., Balfourier F., Bordes J., Greenland A.J., Powell W., Mackay I.J. (2013). *Rht*-*1* and *Ppd*-*D1* associations with height, GA sensitivity, and days to heading in a worldwide bread wheat collection. Theor. Appl. Genet..

[17] Grogan S.M., Brown-Guedira G., Haley S.D., McMaster G.S., Reid S.D., Smith J., Byrne P.F. (2016). Allelic Variation in Developmental Genes and Effects on Winter Wheat Heading Date in the U.S. Great Plains. PLoS ONE.

[18] Bentley A.R., Turner A.S., Gosman N., Leigh F., Maccaferri M., Dreisigacker S., Greenland A.J., Laurie D.A. (2011). Frequency of photoperiod-insensitive Ppd-A1a alleles in tetraploid, hexaploid and synthetic hexaploid wheat germplasm. Plant Breed..

[19] Nishida H., Yoshida T., Kawakami K., Fujita M., Long B., Akashi Y., Laurie D.A., Kato K. (2013). Structural variation in the 5′ upstream region of photoperiod-insensitive alleles Ppd-A1a and Ppd-B1a identified in hexaploid wheat (*Triticum aestivum* L.), and their effect on heading time. Mol. Breed..

[20] Seki M., Chono M., Nishimura T., Sato M., Yoshimura Y., Matsunaka H., Fujita M., Oda S., Kubo K., Kiribuchi-Otobe C. (2013). Distribution of photoperiod-insensitive allele Ppd-A1a and its effect on heading time in Japanese wheat cultivars. Breed. Sci..

[21] Bonvicini M. (1936). Genealogical Selection of the Wheat "Solina".

[22] Bocci R., Bussi B., Petitti M., Franciolini R., Altavilla V., Galluzzi G., Di Luzio P., Migliorini P., Spagnolo S., Floriddia R. (2020). Yield, yield stability and farmers’ preferences of evolutionary populations of bread wheat: A dynamic solution to climate change. Eur. J. Agron..

[23] Casañas F., Simó J., Casals J., Prohens J. (2017). Towards an evolved concept of landraces. Front. Plant Sci..

[24] Raggi L., Pacicco L.C., Caproni L., Álvarez-Muñiz C., Annamaa K., Barata A.M., Batir-Rusu D., Díez M.J., Heinonen M., Holubec V. (2022). Analysis of landrace cultivation in Europe: A means to support in situ conservation of crop diversity. Biol. Conserv..

[25] Badeck F.-W., Rizza F. (2015). A Combined Field/Laboratory Method for Assessment of Frost Tolerance with Freezing Tests and Chlorophyll Fluorescence. Agronomy.

[26] Yan L., Helguera M., Kato K., Fukuyama S., Sherman J., Dubcovsky J. (2004). Allelic variation at the VRN-1 promoter region in polyploid wheat. Theor. Appl. Genet..

[27] Fu D., Szűcs P., Yan L., Helguera M., Skinner J.S., Von Zitzewitz J., Hayes P.M., Dubcovsky J. (2005). Large deletions within the first intron in VRN-1 are associated with spring growth habit in barley and wheat. Mol. Genet. Genom..

[28] Yan L., Loukoianov A., Tranquilli G., Helguera M., Fahima T., Dubcovsky J. (2003). Positional cloning of wheat vernalization gene *VRN1*. Proc. Natl Acad. Sci. USA.

[29] Beales J., Turner A., Griffiths S., Snape J.W., Laurie D.A. (2007). A pseudo-response regulator is misexpressed in the photoperiod insensitive Ppd-D1a mutant of wheat (*Triticum aestivum* L.). Theor. Appl. Genet..

[30] Guo W., Xin M., Wang Z., Yao Y., Hu Z., Song W., Yu K., Chen Y., Wang X., Guan P. (2020). Origin and adaptation to high altitude of Tibetan semi-wild wheat. Nat. Commun..

[31] Ayalew H., Sorrells M.E., Carver B.F., Baenziger P.S., Ma X.F. (2020). Selection signatures across seven decades of hard winter wheat breeding in the Great Plains of the United States. Plant Genome.

[32] R Core Team (2021). R: A Language and Environment for Statistical Computing.

[33] Goudet J., Jombart T., hierfstat: Estimation and Tests of Hierarchical F-Statistics (2022). R Package Version 0.5-11. https://CRAN.R-project.org/package=hierfstat.

[34] Klos K.E., Huang Y., Bekele W.A., Obert D.E., Babiker E., Beattie A.D., Bjørnstad Å., Bonman J.M., Carson M.L., Chao S. (2016). Population genomics related to adaptation in elite oat germplasm. Plant Genome.

[35] Lilin Y. (2022). CMplot: Circle Manhattan Plot. R Package Version 4.2.0. https://CRAN.R-project.org/package=CMplot.

[36] Rizza F., Pagani D., Gut M., Prášil I., Lago C., Tondelli A., Orrù L., Mazzucotelli E., Francia E., Badeck F.-W. (2011). Diversity in the response to low temperature in representative barley genotypes cultivated in Europe. Crop Sci..

[37] Negri V., Maxted N., Veteläinen M., Veteläinene M., Negri V., Maxted N. (2009). European landrace conservation: An introduction. European Landraces: On-Farm Conservation, Management and Use.

[38] Bellon M.R., Dulloo E., Sardos J., Thormann I., Burdon J.J. (2017). In situ conservation—Harnessing natural and human-derived evolutionary forces to ensure future crop adaptation. Evol. Appl..

[39] Würschum T., Boeven P.H., Langer S.M., Longin C.F.H., Leiser W.L. (2015). Multiply to conquer: Copy number variations at Ppd-B1 and Vrn-A1 facilitate global adaptation in wheat. BMC Genet..

[40] Royo C., Dreisigacker S., Soriano J.M., Lopes M.S., Ammar K., Villegas D. (2020). Allelic variation at the vernalization response (Vrn-1) and photoperiod sensitivity (Ppd-1) genes and their association with the development of durum wheat landraces and modern cultivars. Front. Plant Sci..

[41] Bélanger J., Pilling D. (2019). The State of the World’s Biodiversity for Food and Agriculture.

[42] Ceccarelli S., Grando S. (2020). Evolutionary plant breeding as a response to the complexity of climate change. iScience.

