# Supplementary material for: Long-Term In Situ Conservation Drove Microevolution of Solina d’Abruzzo Wheat on Adaptive, Agronomic and Qualitative Traits
Source: Plants (Basel). 2023 Mar 14;12(6):1306. doi: 10.3390/plants12061306 (PMC10057728; doi:10.3390/plants12061306)
Supplement: Supplementary file 1 [file plants-12-01306-s001.zip › plants-2174236-supplementary.pdf]

plants

MDPI

Article

Long-term in situ conservation drove microevolution of Solina d'Abruzzo wheat on adaptive, agronomic and qualitative traits

Caterina Morcia[1], Riccardo De Flaviis[2], Valeria Terzi[1], Maria Eugenia Gasparelli[1], Roberta Ghizzoni[1], Franz-W. Badeck[1], Fulvia Rizza[1], Veronica Santarelli[2], Giorgio Tumino[3], Giampiero Sacchetti [2]*

[1] Consiglio per la Ricerca in Agricoltura e l'Analisi dell'Economia Agraria—Centro di Ricerca Genomica e Bioinformatica (CREA-GB), Via San Protaso 302, 29017 Fiorenzuola d'Arda, Italy
[2] Department of Bioscience and Technology for Food, Agriculture and Environment, University of Teramo, Via R. Balzarini 1, 64100 Teramo, Italy
[3] Plant Breeding, Wageningen Plant Research, Wageningen University & Research, Droevendaalsesteeg 1, 6708 PB Wageningen, The Netherlands
Author to whom correspondence should be addressed.
* Correspondence: gsacchetti@unite.it; Tel.: +39 0861 266977

Supplementary Information

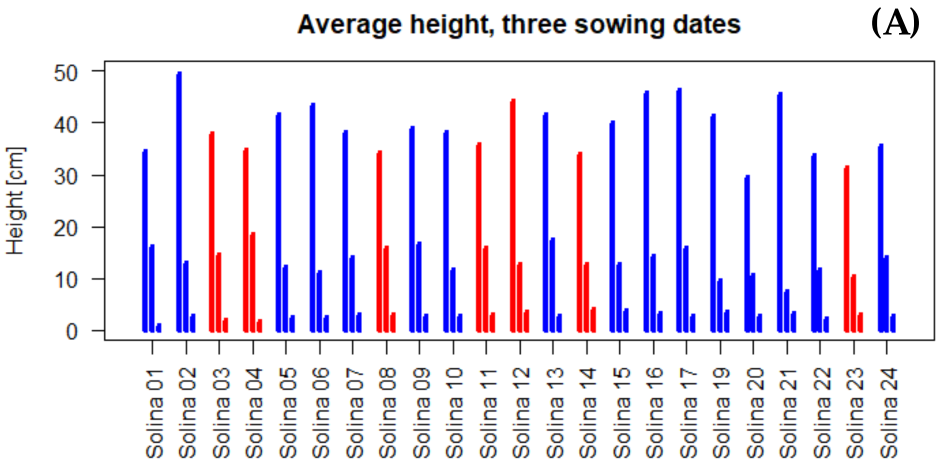

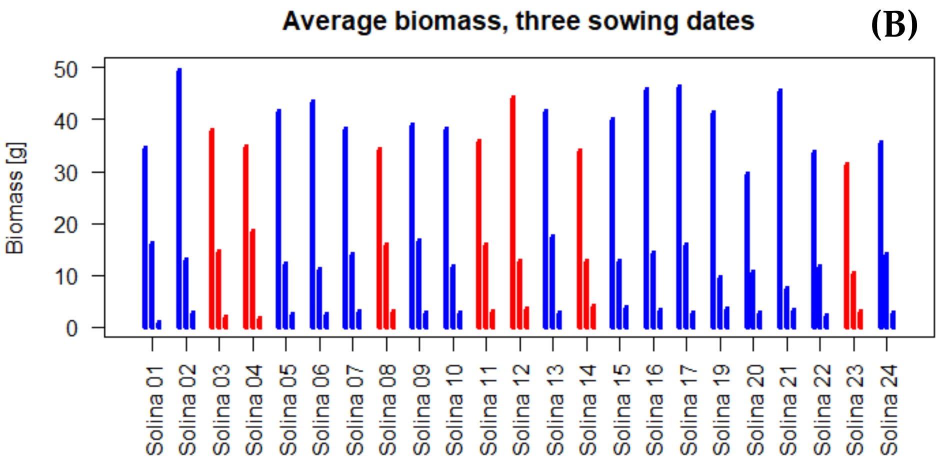

Figure S1. (A), Plant height [cm], (B), Biomass [g], bars from left to right correspond to sowing in November, February, and June, the color of the bars indicates membership to the red and blue clusters according to De Flaviis et al. [5].

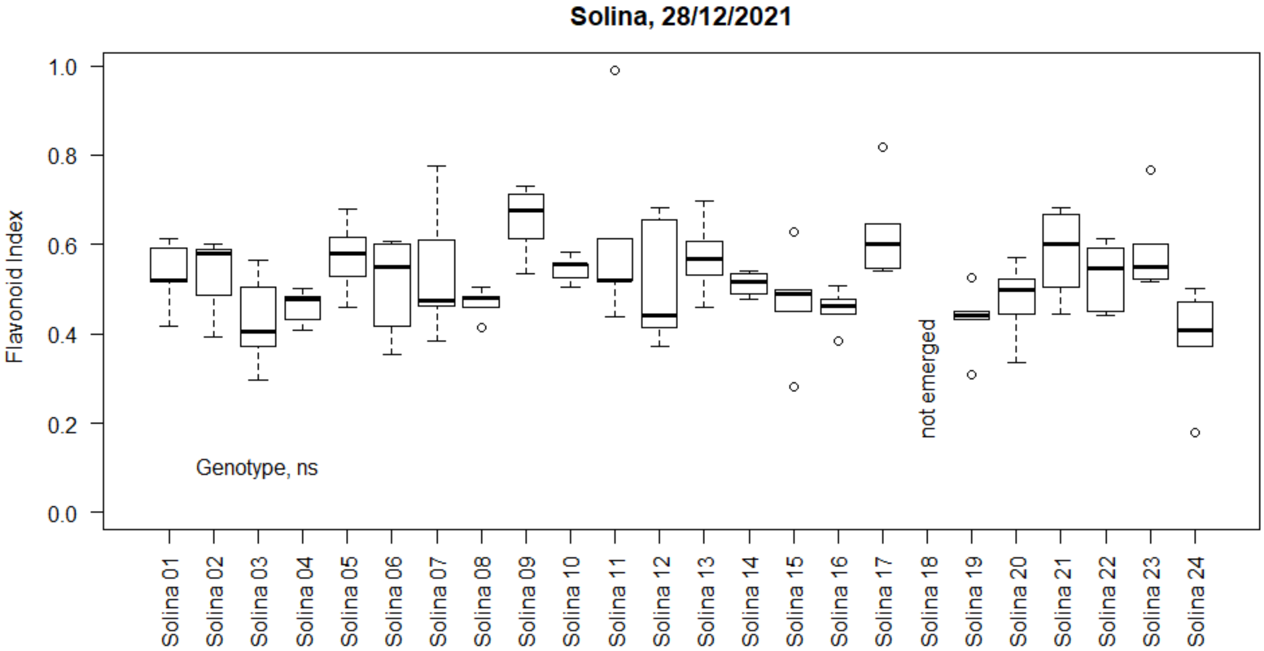

Figure S2. Dualex flavonoid index measured at December 28th 2021, i.e. at DAI 21.

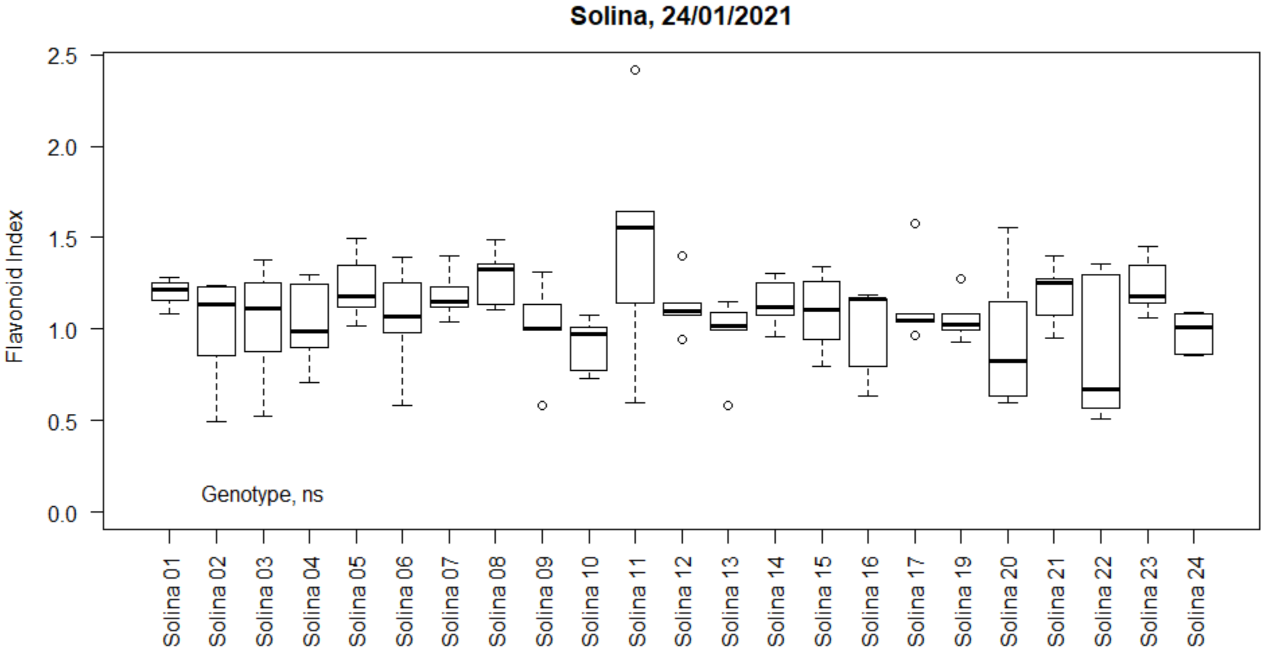

Figure S3. Dualex flavonoid index measured at January 24th 2022, i.e. at DAI 48.

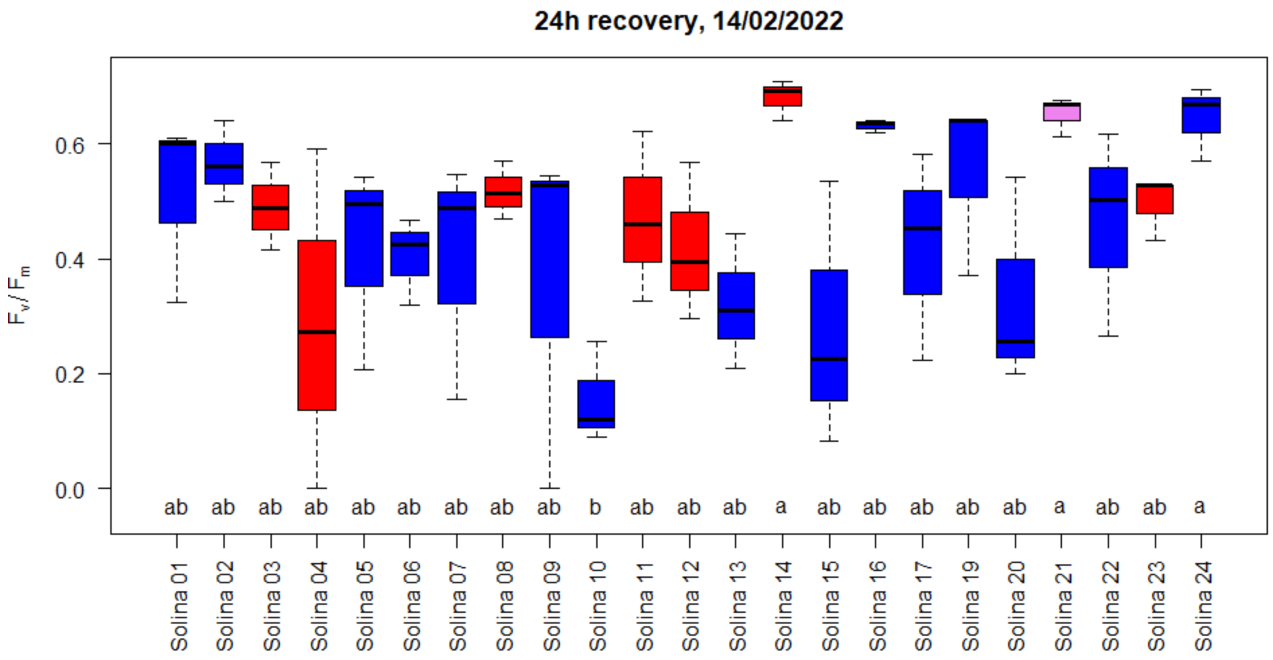

Figure S4. Fv/Fm, an indicator of freezing damage to PSII, measured after freezing stress at −14 °C and 24 h of recovery under acclimation conditions. The first experiment was on cut leaves. The color of the boxes indicates membership to the red and blue clusters, according to De Flaviis et al. [5], with population 21 belonging to the blue cluster but closest to the red cluster shown in purple.

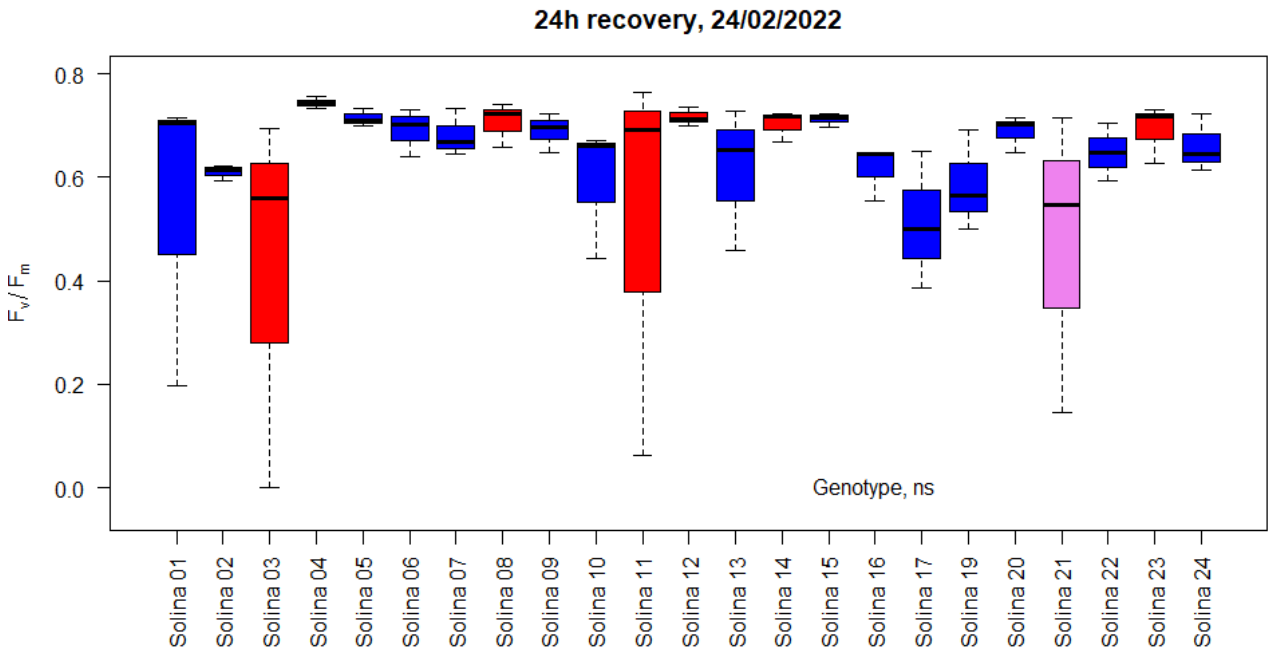

Figure S5. Fv/Fm, an indicator of freezing damage to PSII, measured after freezing stress at −14 °C and 24 h of recovery under acclimation conditions. The first experiment was on cut leaves. The color of the boxes indicates membership to the red and blue clusters according to De Flaviis et al. [5], with population 21 belonging to the blue cluster but closest to the red cluster shown in purple.
